# Inferential schema in Akkadian diagnosis: the case of Ah̬h̬$\bar{a}$zu

**DOI:** 10.1007/s40656-025-00674-6

**Published:** 2025-06-12

**Authors:** Cristina Barés Gómez

**Affiliations:** https://ror.org/03yxnpp24 grid.9224.d 0000 0001 2168 1229 D. Filosofía, Lógica y Filosofía de la ciencia, University of Seville, Sevilla, Spain

**Keywords:** Akkadian medicine, Abduction, Medical reasoning, GW model

## Abstract

The aim of this work is to analyze Akkadian medical diagnosis by examining the reasoning involved in the process. The analysis highlights the importance of uncertainty in the timeline of inference. While prognosis pertains to the future, diagnosis concerns something different; it relates to what has already occurred. It is proposed that the analysis would be incomplete without considering the roles of both the past and present within the inferential framework. Ancient medical diagnosis must be understood by accounting for the entire reasoning structure, which is not captured in a single text, for which reason it is necessary to analyze both the diagnostic and therapeutic kind. This work draws on translations of these texts by Assyriologists. Ancient medical science needs to be studied from multiple perspectives, and the logic and philosophy of science can help to gain a better understanding of its practice and methodology.

## Introduction

Akkadian medicine refers to a collection of medical texts discovered at various archeological sites^1^, the earliest texts dating to around 2000 BC, during the Third Dynasty of Ur (Biggs, 2005). These early texts are written in Sumerian^2^, whereas several later ancient medical texts are written in Akkadian. Although both are inscribed on clay tablets, the languages differ and the writing signs vary across different periods. Sumerian writing is logographic, whereas Akkadian writing incorporates Sumerian logograms along with logo-syllabic cuneiform script.

Regarding the Akkadian medical texts, there are, for example, tablets from Nippur dating to the 18th century BC (Geller, 2006), as well as a range of texts from the first millennium BC, known as the Diagnostic Handbook (Labat, 1951; Heeßel, 2000; Schmidtchen, 2021) (also referred to as the Diagnostic-prognostic Handbook, SA.GIG or *Sakikk*$\hat{u}$ series)^3^. This handbook, consisting of diagnostic-prognostic omens.^4^ serves as a sort of medical guide (Stol 1991-92) that predicts the patient’s future after describing the symptoms of his illness.^5^ The diagnosis typically ends with a prognosis: either “he will die” or “he will live.”

There are also several texts that can be classified as materia medica, which list plants, stones, or diseases, an example of this being BAM 1 (Köcher, 1963-2017). The final text type addressed here is the most common in Akkadian medicine, viz. those known to Assyriologists as “therapeutic texts.” (Thomson, 1923),(Köcher, 1963-2017). These texts, which consist of a combination of recipes, lists of *materia medica*^6^, and incantations, have a distinctive structure typically focusing on symptoms and their treatment, while rarely naming the illness explicitly.

Akkadian medical experts were known as *as*$\hat{u}$ (physician), $\bar{a}$ $\check{s}$ipu/ma$\check{s}$ma$\check{s}$ $\check{s}$u (exorcist), or *b*$\bar{a}$*r*$\hat{u}$ (diviner)(Koch, 2015). In fact, there was no clear distinction between physicians, exorcists, apothecaries, and diviners. Even though it seems that all these specialists approached medical issues from different perspectives, the boundaries between their roles are not clearly defined for researchers^7^.

The study of Akkadian medicine has been approached from various perspectives. A history of science and Assyriology approaches have produced remarkable studies, including translations of texts (Heeßel, 2004), contributing to broaden our understanding of Mesopotamian culture, language, and medicine. Another important approach seeks to understand the concept of disease in ancient Mesopotamia in comparison to modern medicine (Scurlock & Andersen, 2005). In fact, both perspectives regard Akkadian medicine as a scientific practice. Although magic is so deeply ingrained in these medical texts that it cannot be decoupled from them, several aspects reflect what might be called scientific reasoning (Geller, 2010) or an incipient medical science.

A third approach is taken here from a logico-philosophical perspective for the purpose of understanding and tracing the evolution of science through the study of reasoning. This epistemological approach reveals how scientific knowledge has evolved by analyzing its methodology. Specifically, the temporal structure of medical diagnosis, prognosis, and treatment in ancient Akkadian texts is examined by focusing on the role of uncertainty in reasoning.

Drawing from previous works approaching the matter from the two aforementioned perspectives and introducing this third approach, the aim is not to classify medical texts from the standpoint of modern medicine or to study Mesopotamian culture from a purely historical perspective. Rather, the intention is to analyze the reasoning used in medical practice, which should provide new insights into Assyro-Babylonian culture and its understanding of disease—or, more precisely, of science, particularly the medical kind.

In what follows, previous works on the structure of medical diagnosis are approached from a logical perspective, analyzing abductive inference in Akkadian medical diagnosis (Barés Gómez, 2018). The spotlight is then placed on the concepts of uncertainty and the future within the inferential schema by examining examples of Akkadian medical diagnoses, primarily focusing on the prognosis. While the structure of diagnosis largely relates to the future, in the fourth section the role of the past within the inferential schema is explored by explaining the “fill-up” and “cut-down” problems in abductive inferences (Magnani, 2015b, 2001). These concepts pertain to what is called selective abduction (commonly used in diagnostic reasoning, which involves selecting criteria from a number of possibilities) and creative abduction (which formulates hypotheses, including new ones)(Magnani, 2015b, 2001)^8^

The intention here is to understand the temporal structure of medical diagnosis through an analysis of the inferences underlying scientific reasoning. It is contended that this reasoning is associated with action and therefore relates to future prognosis. However, it is also anchored in the past because, even in the absence of evidence, it is linked to hypotheses formulated in relation to past events. The question that triggers the inference is situated in the present, a process known as diagnosis triggering.

This kind of analysis should help to understand how the temporal structure of scientific reasoning develops and its epistemological role. Several Akkadian examples are presented below to illustrate the inferential structure. In the final section, the concept of practical reasoning and how the temporal structure is crucial for ignorance-preserving inference are explained. Uncertainty is a fundamental aspect of such inferences, particularly in Akkadian medical diagnosis.

## Akkadian texts and inferences

Before analyzing inferences in Akkadian medicine, it is essential to explain what an inference is and how it has been studied in Akkadian texts. An inference is simply a relationship between a premise and a conclusion, typically represented as one between $\alpha$ (the premise) and $\beta$ (the conclusion). Generally, when referring to a logical relationship, this signifies a deductive relationship that must be a necessary connection between $\alpha$ and $\beta$. This relationship is sometimes expressed in natural language through a conditional structure, such as “if $\alpha$, then $\beta$.” Following Rochberg (2010), this is the relationship found in Akkadian omens. As she correctly explains, in the statement, “If Jupiter becomes steady in the morning, enemy kings will be reconciled,” the relationship—regardless of how it arises (whether through analogy or otherwise)—constitutes a structure of a deductive process. Thus, when we have $\alpha$, we necessarily have $\beta$.

$$\alpha \rightarrow \beta$$

$$\alpha$$

——-

$$\beta$$

This is a common deductive step known as *modus ponens*. In fact, this structure relates to the future and has the form of a prediction. It is certainly a rational step, as Rochberg (2010) notes, which often appears in scientific reasoning. Of course, it is bold to assert that pre-logical thinking exists when there are predictions that function as completely logical deductive processes.

Nevertheless, deduction is just one way of relating premises and conclusions that seems to appear in omens, but not the only way reasoning occurs. There are other types of inference, such as the inductive and abductive kind. When discussing predictions (as seen in omens), deductions are usually performed, whereas the explanations found in medical texts are associated with other kinds of inference, with abduction being the most prominent. However, these can intertwine with other types of inference,^9^ as will be explained below.

Returning to medical reasoning in Akkadian texts, as already mentioned, there are several kinds. Nevertheless, pride of place should be given to the Diagnostic Handbook (Labat, 1951Heeßel, 2000 ; Schmidtchen, 2021), an important compendium of medical diagnoses from Antiquity. The structure of Akkadian medicine is divided into signs/symptoms, diseases, prognoses, and treatments. The Diagnostic Handbook primarily addresses signs/symptoms, illnesses, and prognoses, while sometimes referring to treatments as well. These texts discuss the causes of diseases or the names of illnesses. Although in most cases the causes appear to be attributed to supernatural entities—such as the well-known “Hand of the God”—there are instances in which the focus is more on the symptoms.^10^ They typically follow this structure^11^:

If (in this or that frame of reference) symptom A (B-Z) is present:

diagnosis (disease name and/or disease causative agent) - justification for disease -

instruction to exorcist - prognosis(es)

The first sentence of the structure in Akkadian medicine corresponds to what Schmidtchen (2021) calls the “topicalisator.” It expresses the preceding circumstances that lead to the diagnosis, which is referred to in abductive reasoning as the “surprising fact/facts.” This structure is now examined while considering the medical reasoning framework and setting aside the prognosis for the moment. The structure of a diagnosis in Akkadian medical texts can be rationally reconstructed as an inferential structure known as “abduction”,^12^ which is an inference that is neither induction nor deduction. Its first complete analysis can be found in Peirce (1931) [CP 5.189] and is schematized as follows:

The surprising fact C is observed.

But if A were true, C would be a matter of course.

Hence, there is reason to suspect that A is true.

This reasoning involves the provisional adoption of a hypothesis and its application in future actions.^13^ This can be illustrated with an example of medical diagnosis from an Akkadian text. The following paragraph comes from the Diagnostic Handbook^14^. Tablet 33 is the only one in the Diagnostic Handbook that uses the correct name of the illness. It should be considered that in ancient medical tracts, signs/symptoms and names are often mixed. Nevertheless, the existence of a tablet with a specific name indicates there is a clearer inferential schema that differentiates between these aspects. Taking a broader perspective is a good starting point when the idea is to explore the schema of reasoning. Thus:

### Example 1

Diagnostic Handbook: possible jaundice. SA. GIG/*Sakikk*$\hat{u}$33/93^15^

Translation: *“*[If his face] is yellow and the inner part of his eyes is yellow (and) the base of the tongue is black, [it is called], [*a*h̬h̬$\bar{a}$*zu*]*”.*

If the signs/symptoms are considered as $\beta$ and the illness as $\alpha$, a structure similar to the following might be obtained^16^:

The patient has the signs/symptoms $\beta$ ([his face] is yellow and the inner part of his eyes is yellow (and) the base of the tongue is black)

If the patient has illness $\alpha$ ([*a*h̬h̬$\bar{a}$*zu*]) $\rightarrow$ then he will have the signs/symptoms $\beta$ ( [his face] is yellow and the inner part of his eyes is yellow (and) the base of the tongue is black)

___________________________

It can be concluded that the patient has illness $\alpha$ ([*a*h̬h̬$\bar{a}$*zu*])

This inferential structure is frequently expressed by a conditional in Akkadian ($\check{s}$*umma*), usually with DIŠ following a list of symptoms (if the patient has any...)^17^ in the protasis and a diagnosis in the apodosis^18^. The physician has the signs/symptoms for an sick person (*marṣu*) in the protasis, along with some contextual data that trigger the inference and serve as the premises. In logical terms, they are typically referred to as the antecedent and consequent of a conditional. Nevertheless, this natural language conditional reverses the deductive inference. The structure consists of premises (protasis) and a conclusion (apodosis).

From a logical perspective, it is necessary to analyze the relationship between these premises (signs/symptoms) and this conclusion (diagnosis/cause).^19^ This is not a deduction because, in the natural language conditional, the antecedent is normally the consequence of an illness. In fact, a diagnosis—the cause—comes first and the signs/symptoms are the consequence of the cause, as shown in the schema presented above. Conversely, in the linguistic conditional, first there are the signs/symptoms and then the consequence as the cause. This inference is not a deduction because, from a deductive point of view, backward reasoning is involved.

## Abductive inference in diagnosis: G-W schema

In Akkadian the conditional demonstrates a backward inference in search of the cause. It is not a *modus ponens* structure because it does not involve forward reasoning typical of an omen, but backward reasoning in the quest for an explanation, even though these two types sometimes employ the same conditional in the Akkadian language. Indeed, if this structure—the one in the Akkadian medical conditional that seeks the causal agent (diagnosis) is accepted—it could be claimed that this is a deduction; however, this inference is invalid. The truth of the premises does not imply the conclusion. Since Aristotle, this has been referred to as the “fallacy of affirming the consequent,” resembling *modus ponens*, but in reverse. When examined from a deductive point of view, it is possible to identify an error in reasoning. This is not a valid argument schema in deductive logic.

$$\beta$$

$$\alpha \longrightarrow \beta$$

———

$$\alpha$$

But is it really an error? While it may not be classified as one, the interesting point here is the fact that this is no longer a deduction but rather an abduction, which is more complex than the fallacy of affirming the consequent. Therefore, the formalization presented above is a simplification of the actual reasoning involved.This form of reasoning does not use the same standards of validity as deductive reasoning, while its correctness resides in its pragmatic virtue.

The GW model, developed by Dov M. Gabbay and John Woods in Gabbay and Woods (2005); Woods (2013), is useful for analyzing this structure. This model of abduction is conceived in terms of interactive constructions and draws from conceptual insights (Magnani, 2017) deriving from the recent revival of the Peircean pragmatic view. That is why the models proposed by Gabbay and Woods (2005), Woods (2013), and Magnani (2017), which understand abduction in terms of cognitive, economic, and ecological considerations, serve as a starting point for practical reasoning in medicine. Woods ((Woods, 2013), p. 376) defines abduction as an “ignorance-preserving” inference, which arises in response to an ignorance problem. A question to which an agent has no answer acts as a cognitive irritant, prompting the formulation of a hypothesis that may serve as the basis for new actions, despite the persistent state of ignorance.

Let us briefly consider the aforementioned example in a GW model (Gabbay & Woods, 2005; Woods, 2013) of abduction, which captures more features than the schema presented earlier:

1. $T!Q(\alpha )$ This corresponds to a *T* (target) resulting from a *Q* (question) that will be answered by $\alpha$, which means that there is an initial question that needs to be answered, which is as follows: from which disease is the patient suffering? In this state of ignorance there is a fact (that the patient is unwell) and it is essential to know what has triggered these signs/symptoms^20^.
2. $\sim (R(K,T))$ [Fact] This surprising fact of the signs/symptoms is not in relation *R* to target *T* that will answer the question and the current state of knowledge *K*. In other words, the reason behind the patient’s sickness is unknown.
3. $\sim (R(K*,T))$ [Fact] There is no successor of knowledge $K*$ in the physician’s current background knowledge. Nor is there any relation *R* between the successor of knowledge $K*$ and target *T*. There is nothing in the physician’s current knowledge that directly indicates what disease the patient has.
4. $H\notin K$ [Fact] In order to answer the question, a hypothesis *H* that does not form part of background knowledge *K* is required. In other words, the physician’s hypothesis about the illness is not to be found in his current knowledge of the patient’s state.
5. $H\notin K*$[Fact] Hypothesis *H* is not in a successor of background knowledge $K*$. Nor can the physician encounter the hypothetical disease in a successor of his current knowledge.
6. $\sim R(H,T)$ [Fact] So, there is no relation *R* between hypothesis *H* and target *T*. Nor is there any previous relation between the illness and the answer to the question.
7. $\sim R(K(H),T)$ [Fact] There is no relation between background knowledge *K*, hypothesis *H*, and target *T*. Nor is there a real link between the illness, the physician’s current knowledge, and his answer.
8. $H\rightsquigarrow R(K(H),T)$ [Fact] A hypothesis *H* is conjectured which has a subjunctive relation $\rightsquigarrow$ in order to interrelate background knowledge *K*, hypothesis *H*, and target *T*. That is, if the physician were familiar with the disease, he might be able to answer the initial question, for it would allow him to explain the signs/symptoms.
9. *H* satisfies conditions $S_{1},...,S_{n}$ [Fact] This hypothetical disease needs to fulfill some conditions to be plausible for the time and place and so forth.
10. Then, *C*(*H*) [Sub-conclusion, 1-7] The physician conjectures the hypothetical disease. At this point, in medicine the hypothesis is usually clinically tested. There are subsequently three possibilities. The first is that the hypothesis is confirmed, signifying that a partial abduction has been performed because the result is no longer a hypothesis^21^. The second is that this is not the case, signifying that the physician has to start from scratch. The third is that neither has the hypothesis been confirmed nor has it been clinically tested. Rather, the physician acts in consequence, treating the patient in order to cure the disease that he has hypothesized. The last possibility leads to step 11.
11. Then, $H^{C}$ [Conclusion 1-8] The physician uses the hypothesis in an ignorance-preserving way. He does not prove it, but acts in consequence by treating the patient. This step, which is frequently achieved in general clinical medicine because material resources are limited, also occurs in emergency situations (Barés Gómez & Fontaine, 2021). In Akkadian medical diagnosis, this step is linked to a prognosis.

The main point that should be highlighted in this schema of abduction is that it is triggered by a surprise; it is an act of guessing, an inkling rather than knowledge. It is also a matter of course^22^ and leads to action. “Abduction surprises are agenda-motivating cognitive irritations,” in Woods’ words (Woods, 2013, p. 366).

To this G-W schema should be added the concepts of selective and creative abduction, as introduced by Magnani in Magnani (2001), which correspond to steps 8 and 9. These types of abduction are quite important for diagnosis because selection involves choosing between hypotheses used in a diagnosis to differentiate among illnesses in a compendium. Creative abduction, on the other hand, occurs when a new hypothesis is formulated. These cases are explored below with Akkadian examples.

## The future: prognosis and uncertainty within the inference schema

Examining the first Akkadian example from section 1, it is possible to identify steps 1 to 10 of the G-W schema of abduction. This is what is referred to as partial abduction in Gabbay and Woods (2005); Woods (2013). The reason for this designation is that the hypothesis is not used for further actions. Medical texts are typically designed for practical situations, while it is common in medical reasoning to consider the hypothetical disease as the starting point in the healing process.

In the present context, practical situations refer to the reasoning that is often applied in medical practice to take action to cure a patient. However, this practice can be qualified in the case of Akkadian texts. Although the purpose of the extant texts still is not fully understood, some authors suggest they were used in medicine, whereas others argue they were merely scholarly copies for training purposes. Arbøll (2019) posits that they might have been written for ritual use, as evidenced by texts from the library of *Kiṣir-A*$\check{s}\check{s}$*ur*.^23^ However that may be the structure of this kind of reasoning is linked, in some way, to practice.

This does not imply that healing must be achieved through modern medicine. In fact, it does not matter whether it is accomplished through pharmacology, dietary recipes, or even rituals, for the inferential schema remains the same. Examining other occurrences of *A*h̬h̬$\bar{a}$*zu* as jaundice in the Diagnostic Handbook, it can be seen how this reasoning continues to be used to certain extent. Prognoses are typically included as well.

### Example 2

Diagnostic Handbook: possible jaundice.SA.GIG/*Sakikk*$\hat{u}$ 22/16^24^

Translation: “If a person’s spittle flows when he speaks, *a*h̬h̬$\bar{a}$*zu* fills his face (and) his bowels are loose, “hand” of curse; he will die.”

In this text, it can be observed how the hypothesis is a matter of course, in Peirce’s words, because it leads to a prognosis. In fact, the prognosis is of utmost importance in Akkadian medicine, as a favorable one guides the physician to a treatment. The pharmacological recipes and rituals are primarily, though not exclusively, found in the therapeutic texts. Conversely, if the prognosis is negative—“he will die”—no treatment is administered. In both cases, the prognosis is the consequence that arises from the hypothesis and directs the physician toward further action.

However, the real activation of the hypothesis occurs when the physician has the prognosis and decides on whether or not he should proceed with treatment. It is in these instances, which the G-W model refers to as full abduction, that the activation of the conjecture in step 11 can be encountered. This subsequent action pertains to future decisions, which is why two aspects specific to the future time structure within the inference schema should be highlighted: first, the role of the prognosis; and second, the role of the hypothesis and its associated uncertainty that leads to a potential treatment. Both of these represent distinct future-time aspects in medical diagnosis.

As already observed, prognosis is fairly important in Akkadian medical diagnosis,^25^ for it addresses future aspects of medical reasoning. Indeed, the patient will only be treated if the prognosis is positive. The next step in the medical diagnosis process for curable illnesses is to treat the patient.

Usually, the prognosis is made in relation to the signs and symptoms, as well as the hypothesis. However, is it possible to determine whether this prognosis should be based on the signs and symptoms or on the hypothesis? The relationship between signs/symptoms and hypotheses can often be rather hazy in medical diagnosis.

### Example 3

Diagnostic Handbook: Sick lung. *SA.GIG/Sakikk*$\hat{u}$ 40/29^26^

Translation: “If an infant’s flesh is unevenly colored with yellow, his insides are cramped, his hands (and) feet are swollen, he has lots of *li’bu* and the lung are sick, “hand” of god; he will get well.”

This text includes the well-known “Hand of” and an illness originating from the affected part of the body. Labat (1951) mentions that this entry may form part of the symptoms rather than an illness per se. The illness is sometimes named after the organ and the line separating signs/symptoms from illness can be rather vague in ancient medicine. Occasionally, the name of the illness refers to its most prominent symptoms, as in the case of “Sick Lungs.”

Does this mean there is no distinction between illnesses and signs/symptoms? It is possible that the different components of the inferential schema in this medical reasoning were not as separate as they are today. Nevertheless, they are somewhat distinct, as evidenced by the differences between diagnosis, signs/symptoms, prognosis, and therapy. In the inference schema found in therapeutic texts, a combination of symptoms is usually provided in the section where the name of the illness is listed. In both types of texts, the aim of the hypothesis is clearly oriented toward the future, but it sometimes appears as the name of the illness and sometimes as the affected body part or as a combination of symptoms.

The difference between the name of the illness and the signs/symptoms is also noted by Schmidtchen (2021).^27^ The diagnoses appearing in the Diagnostic Handbook specify the affected part and are considered as such because they appear in the apodosis. This structure is not actually that different from the prognosis structure in Hippocratic medicine (Hipócrates, 2002 Hipócrates, 2016), where the prognosis predominated. Rather than implying that there is no diagnosis at all, this is more general (sometimes referred to as a general pathology), while the most detailed aspects are the prognosis and semiotics (the study of signs/symptoms). It is sufficient to consider the prognostic (Hipócrates, 2016) or epidemics (Hipócrates, 2002), which refer to pathology in general terms and emphasize signs/symptoms. In any case, the predictions arising from the hypothesis or semiotics (i.e., the prognosis) should be followed, before deciding whether to treat the patient accordingly.

The prognosis^28^, as already noted, is fundamental in Akkadian medicine because it is linked to an action. The development of a prognosis, one of the most important aspects of medical methodology, derives from the hypothesis and systematic observation of signs/symptoms. As noted above, in Akkadian medicine, as in the Hippocratic kind, there can sometimes be a gap between semiotics and prognosis. In scientific methodology, this is a crucial element being subsequently referred to as empirical medicine (Bernard, 1966).

In the Diagnostic Handbook, the diagnosis and prognosis are based on various factors, including blood vessels, temperature, sensations of pain, color changes, the weather, fevers, temperature fluctuations, and diet.^29^ They are also associated with time (explicitly mentioning the duration of the illness), age, gender, social class, and other factors. In this part of the text, the well-known concept of crisis, which can also be found in Greek (Hipócrates, 2016, Hipócrates, 2002) and Egyptian medicine (Edwin Smith Papyrus (Breasted, 1930)) is often encountered.

### Example 4

Diagnostic Handbook: SA.GIG/*Sakikk*$\hat{u}$ 3/79^30^

Translation: “If in his head he burns with fever (and) over the course of a day it leaves him and then (later) it overpowers him (and) flows over him for two days (as in ) affliction by [a ghost] and the plantar surfaces of his feet (feel) cold, (and) when (the fever) releases him, his hands and his feet are hot as in affliction by a ghost, *a*h̬h̬$\bar{a}$*zu*, [if] (the course of the fever ) is regular, [he will get well].”

This example includes a scientific methodology that emphasizes the systematic observation characteristic of Hippocratic medicine^31^. The most important components are the semiotics, or signs/symptoms, plus the prognosis that can be derived from them. Although the example also includes the hypothesis or illness, the key aspect is systematic observation, which Claude Bernard refers to as empirical medicine (Bernard, 1966).

Despite the fact that Hippocrates is often credited with developing the theory, from a scientific methodology perspective the most significant element is not the “theory of humors” but rather the methodology of observation found in the prognostic texts, which is also inherent to Akkadian medicine, as clearly reflected in the *Sakikk*$\hat{u}$.

The future focus of the prognosis—the prediction of what will follow the hypothetical disease (Barés Gómez & Fontaine, 2022)—is followed by an action. As already seen, the prognosis determines whether treatment will be implemented or not, which leads to the next aspect relating to the future in medical reasoning, viz. the treatment.

The Diagnostic Handbook follows the structure of signs/symptoms, illness, and prognosis, which constitutes a full abduction due to the way the prognosis is linked to an action. It maintains the classic structure of omens: “if such and such, then such and such (may result),” indicating whether the patient will live or die.^32^ The treatment is described in detail in what Assyriologists refer to as therapeutic texts.^33^ These specific texts have a different structure focusing on this aspect. In fact, they often include a structure that presents signs/symptoms and the corresponding treatment. They also typically begin with an “if” clause, defining the symptoms as “if the patient suffers from a certain disease, syndrome, or signs/symptoms,” before providing recipes with instructions on what to do “in order to cure him,” leading to the conclusion that “he will get better” or “he will improve”^34^. The following exemplifies the structure of the occurrence of *A*h̬h̬$\bar{a}$*zu* in a chapter concerning the stomach, specifically in tablet 3 of the seventh subseries of the Nineveh recension of UGU.^35^

### Example 5

BAM 578 (iv 26-27)^36^

Translation: “If a person’s flesh is yellow, his face is yellow and black (and) the base of his tongue is black, it is called *a*h̬h̬$\bar{a}$*zu.* You grind a large steppe-dwelling* pizzalurtu* - lizard (and) you have him drink (it mixed) with beer. The *a*h̬h̬$\bar{a}$*zu* inside him should be excreted.”

This example illustrates the structure commonly found in therapeutic texts which tend not to refer explicitly to the illness, although it is noted in this case. This provides a different inference structure. Indeed, the entire inference structure, resulting in a full abduction, complete with the activation of the abduction and a prediction, can be observed in the following schema:

Diagnosis: Signs/symptoms - surprising fact

Illness - Hypothesis - Cause

Prognosis - further consequence of the hypothesis which leads to treatment or otherwise. It might be seen as an activation of the hypothesis and a prediction.

If it is treatable.

Therapeutics: Returning to the signs/symptoms (which sometimes refer to an illness), the physician examines the illness again through the diagnostic texts and in terms of a positive prognosis.^37^

Treatment - activation of the hypothesis which follows on from the prognosis

This structure could reflect, as some Assyriologists have suggested, that the diagnostic texts served as a guide for their therapeutic counterpart (Stol 1991-92).^38^ The primary difference between the two types of texts is that the diagnostic ones specify the etiology, whereas the therapeutic ones do not.^39^ The first sentence of example 5 is a more or less verbatim citation from the Diagnostic Handbook; specifically, it seems to combine elements from SA-GIG 33:92 and 93^40^. The structure of the diagnostic texts consists of semiotics, etiology, and prognosis, whereas the therapeutic texts begin with the prognosis. As a matter of fact, it could be assumed that the etiology was already known and had derived from the diagnostic texts. The therapeutic texts focus on one aspect of medical diagnosis, namely, its practical application,^41^ which involves the activation of the hypothesis and the treatment following a positive prognosis (or a negative one with some chance of improvement), as well as an experimental component dedicated to trial and error.^42^

However, it is important to note that researchers have only occasionally found correlations between the two types of texts.^43^ Do these infrequent correlations imply that there is no relationship between them and hinder our understanding of medical inference in both texts as a whole? Does it suggest that the diagnostic texts offer a clear inference aimed at revealing the sources through abductive reasoning, whereas the therapeutic texts only present what could be called symptomatic treatments or empirical evidence of various treatments? If so, these latter texts would not align with the norms of scientific diagnostic reasoning in medical science. Nonetheless, this raises a common question in the philosophy of medicine. The answer may resonate with what Claude Bernard referred to as the experimental idea (Bernard, 1966; Barés Gómez & Fontaine, 2022). If one lacks the diagnostic-prognostic component and cannot engage in trial and error, what exactly is one attempting to test?^44^ Even if it is impossible to detect a precise correlation between these two elements, both are necessary for scientific medical reasoning^45^

Regarding the treatment of jaundice, there are two terms that the ancient Babylonians used to describe this condition. The first is *amurriq*$\bar{a}$*nu*, which seems to refer to a milder form of jaundice and is attributed to the Hand of Gula.^46^ The second term is *A*h̬h̬$\bar{a}$*zu*, also known as the Catcher-demon (or the demon that causes jaundice), which was associated with the “Hand” of Gula’s divine consort, Ninurta. Typically, the consequences of both conditions are considered to be serious for the patient. However, there are treatments for jaundice, including the use of *bu’*$\check{s}\bar{a}$*nu* and *li*$\check{s}\bar{a}$*n kalbi* (literally “dog’s tongue” plant), as attested by Böck (2014).

### Example 6

BAM VI 578 iv:17^47^

If a man eyes are full with jaundice: Crush the *bu’*$\check{s}\bar{a}$*nu* plant, let him drink it with beer and then he shall recover.

Assyriologists typically refer to the $\bar{a}\check{s}$*ipu*—the practitioner who works with diagnostic texts—as the exorcist and the *as*$\hat{u}$—the one who works with therapeutic texts—as the real physician. There are differences of opinion on this matter, this distinction seemingly becoming blurred as more is learnt about Mesopotamian medicine. Nevertheless, the most important aspect that should be underscored is the practical proof of the remedies, namely, the trial-and-error aspect of empirical medicine from a methodological perspective, specifically concerning pharmacological treatment^48^. The magical elements of the diagnostic texts appear to fall outside scientific methodology.

However, from an inferential and philosophical standpoint in medicine, the entire inferential structure is required to discuss proper scientific methodology. Otherwise, the first aspect, associated with the diagnostic texts, only provides semiotics (systematic observation, which is the most empirical component of medical reasoning) in some tablets and pure speculation in others (such as the diagnostics associated with the “Hand of”). In the second case, the therapeutic texts, without the *Sakikk*$\hat{u}$, are merely symptomatic treatments deriving from trial and error, lacking any scientific planning. To fully understand medical reasoning in Akkadian texts, these texts must be considered as a cohesive whole.

Another aspect that merits consideration is that, even though the term “therapeutics” is normally used when referring to the therapeutic texts, in the *Sakikk*$\hat{u}$ reference is also made to therapy.

### Example 7

Diagnostic Handbook: SA.GIG/S*akikk*$\hat{u}$ 31/1-2^49^

Translation: “[If *ṣētu* burns a person] and on the day (you see the patient), he has chills, that person has been sick for three days; [in order that] [his] [illness] not be prolonged, if you repeatedly rub him gently with hot oil and first quality beer, he should recover.”

This is a proper prognostic text in ancient medicine indicating a specific number of days, which comes from the $\check{s}$*umma* ṣē*tu i*h̬*mussuma* of the Diagnostic Handbook, not the therapeutic texts. Once again, in terms of scientific medical reasoning, there is a more complete schema in the diagnostic texts than in the therapeutic ones. Similar to Greek medicine, the medical reasoning has the structure of signs/symptoms (semiotics), which is established in empirical medicine through systematic observation, as emphasized by Hippocrates. In fact, several Assyriologists, such as Heeßel (2024a), recognize that it is likely that the Diagnostic Handbook has an empirical basis that the historical omens lack. The medical omens seem to be based on real observations of signs/symptoms, representing the triggered aspect of the reasoning and the empirical foundations of scientific reasoning.^50^

When examining the subsequent part of the reasoning—specifically, the cause of the disease—which in Greek medicine is primarily found in Galen and the theory of humors, a similar element in Akkadian medicine can be observed. This cause or hypothesis embodies the experimental idea, in Bernard’s words (Bernard, 1966), leading to trial and, subsequently, a prognosis.^51^ Empirical medicine, as science, is developed through Hippocratic prognosis and sometimes bypasses the underlying cause.

The most important aspect is the development of systematic observation and the prognosis that arises from it, because it is grounded in empirical facts, whereas the causes in Galenic medicine are often less clear. The lack of emphasis placed on the causes of illness in Hippocratic medicine is frequently discussed, as it tends to establish a general pathology. Even when compared to Greek medicine, the Diagnostic Handbook contains what is clearly empirical medicine—as in the last text mentioned above—whereas other texts focus on the causes of illnesses described in Galenic medicine.

Ergo, elements of scientific medicine can be observed in both traditions, specifically, empirical medicine and the exploration of causes. The crucial factor in developing scientific medical methodology is not the random trial and error present in therapeutic texts, but rather the systematic observation that leads to hypotheses and prognoses. Trial and error is ineffective without a guiding hypothesis. These hypotheses emerge from an inferential step (abduction), transitioning from a set of empirical facts (systematic observation: signs/symptoms) to the hypothesis (cause/illness) and subsequently activating treatment through action.

These are the scientific foundations of medical reasoning, as reflected in the Diagnostic Handbook. Moreover, there are also tablets, such as no. 31, which include recipes, indicating a complete structure that incorporates trial and error in the treatment process. In fact, as mentioned earlier, in Akkadian medicine, treatment is associated with a positive prognosis, as evidenced by tablet *Sakikk*$\hat{u}$ 31/1-2. In the Diagnostic Handbook, treatment is administered for cases of overheating with the aim of preventing a prolonged illness.

Treatment plays an important role in the schema. There is a full abduction with post-confirmation (confirmation following full abduction) if the proper treatment for the illness is determined. In any case, the uncertainty or ignorance stemming from the hypothesis persists when deciding on a course of treatment. This ignorance is only partially resolved after the hypothesis is employed and the reaction can be observed following the prognosis and treatment, but the hypothesis must be used to achieve this.

This uncertainty not only remains tied to the future, leading to an uncertain outcome, but also connects to the past. While the future is reflected in the prognosis and treatment, along with the aspects of uncertainty that arise directly from the hypothesis, there are also links to the past through the diagnosis. This leads to the concepts of selective or creative abduction as discussed by Magnani (Magnani, 2001, 2015a, b, 2017, 2019a).

## The role of the past: diagnosis and uncertainty

Medical diagnosis is usually associated with the future because the goal is to cure the patient over time. The medical diagnosis has a reasoning structure that results in further action for it is a practical reasoning aimed at activating a hypothesis at a future moment. Nevertheless, are healing and death solely related to the future or does the past play a role in them as well?

The past is important because an attempt should be made to isolate the possible causes (Chiffi, 2021). The essential point here is that the inference schema used in medical diagnosis also has links to the past regarding the hypothesis of the illness, which may involve selection or creation, as explained by Magnani (Magnani, 2001, 2017, 2019b). Until now, the focus has been placed on step 11 of the GW model, which describes the future activation of the hypothesis. The time has now come to turn our attention to the issues associated with steps 8 and 9 of the GW model.

- 8. $H\rightsquigarrow R(K(H),T)$ [Fact] So we conjecture a hypothesis *H*.
- 9. *H* satisfies conditions $S_{1},...,S_{n}$ [Fact]

Abduction is considered here as a way of formulating plausible hypotheses that serve to move forward. This type of formulation involves choosing or selecting hypotheses from those available. In fact, their selection, at least in medicine, connects with the practical aspects of the field. The disease is selected from a compendium of known conditions. When examining Akkadian medical diagnosis, it can be observed that this is precisely the function of some tablets from the Diagnostic Handbook. This is one of the earliest compendia in the history of medicine, whose importance lies in how the texts identify known maladies and their signs and symptoms. These texts, which might have served as manuals for students or apprentices, reflect general practice, rather than a random and isolated remedy for somatic treatment. Nevertheless, this step, which is not straightforward, has been a topic of debate in abductive studies.

There are two movements in steps 8 and 9. First, there is a process that is referred to as the “fill-up problem,” as described by Magnani (Magnani, 2001, 2015b, 2019b). It is first necessary to determine how to fill up the plausible hypotheses and according to which criteria. I believe there is no clear fill-up criterion for the possible hypotheses because the usual criteria mentioned in this context are neither sufficient nor necessary for formulating a satisfactory one. In point of fact, a criterion of plausibility, minimality or consistency is often mentioned. However, none of these criteria are entirely satisfactory because the hypothesis might not be minimal, could be contradictory, or may even be implausible (Barés Gómez & Fontaine, 2021). None of these could serve as a definitive criterion for supporting the hypothesis. The most important scientific theories frequently fall outside these criteria.

Even without clear and definitive criteria, plausible hypotheses can still be filled up, this aspect being linked in medicine to the past in the sense that those hypotheses are filled up with known maladies. Future reasoning is based on past reasoning. Thus, the selection problems, which are the focus of what is called selective abduction, arise in the diagnosis when choosing an illness from a compendium of known maladies, such as the Diagnostic Handbook in Akkadian medicine.

After filling the gap, there are several possibilities to choose from. This aspect is referred to as the “cut-down problem” in abduction (Magnani, 2015a, b). Again, the issue of criteria crops up: are there criteria to cut down the plausible hypotheses? The answer is “no”. While possibilities could be discussed, in some cases the most improbable option is chosen due to the associated risks.^52^ The following could be considered as an example of these movements in Akkadian medicine:

### Example 8

Diagnostic Handbook: SA.GIG/S*akikk*$\hat{u}$ 13/75-76^53^

Translation: “If he was injured on his abdomen (and he has been sick for ) nine days, ’hand’ of the twin gods. If (it is) second (day), ’hand’ of *Adad*. If the third, ’hand’ of *Ea*. If the fourth, ’hand’ of *Dingirmah*h̬. If the fifth, ’hand’ of *Papsukkal*.”

In these texts, there are several diseases that could be considered more or less likely based on the signs and symptoms. This process is often referred to as differential diagnosis^54^; however, it is primarily about choosing among possibilities, a process that is also described in other tablets. In fact, the fill-up movement has already been established, but the cut-down movement is not as clear, and the physician often leaves the possibilities open or attempts to choose among them. This aspect also highlights the defeasible nature of the hypothesis and its merely conjectural status. It is a conjecture anchored in the past but intended for use in the future.

This kind of selection between plausible hypotheses is quite common in medicine. Nevertheless, it implies a broad knowledge of medical science and disease categories. Another question that should also be posed is what happens when the disease is unknown and cannot be found in the handbook. This situation also connects with the past, but in the sense that the disease was never considered. This case is what is referred to as creative abduction (Magnani, 2001, 2015b, 2017).

Creative abduction occurs when, as in our medical case, the hypothesis is not a known disease. The physician creates a new syndrome because the signs and symptoms do not match any known illness. As mentioned in Scurlock and Andersen (2005), there are some syndromes in ancient Akkadian medicine that were associated with demons. Considering the way in which illnesses were categorized as gods, ghosts, or demons, the creation of a new syndrome also involved that of a new divine figure. There are several examples in Akkadian literature that might reflect this, such as *Lama*$\check{s}$*tu*, *ah̬*h̬$\bar{a}$*zu*, *mi*$\check{s}$*ittu,* and *al*$\hat{u}$^55^. Following Adamson (1993), *a*h̬h̬$\bar{a}$*zu* is described as a symptom of jaundice or a demon personifying jaundice itself. As mentioned earlier, there is another term that describes jaundice: *amurrīq*$\bar{a}$*nu*. Some texts attempt to differentiate between the two. Here is a final example from Akkadian medical diagnosis of this *a*h̬h̬$\bar{a}$zu, which some authors consider to be a possible creation^56^ and, in inferential terms, can be seen as a case of creative abduction.

### Example 9

Diagnostic Handbook:SA.GIG/*Sakikk*$\hat{u}$ 3/79-80.^57^

Translation: “If in his head he burns with fever (and) over the course of a day it leaves him and then (later) it overpowers him (and) flows over him for two days (as in) affliction by [a ghost] and the plantar surfaces of his feet (feel) cold, (and) when (the fever) releases him, his hands and his feet are hot as in affliction by a ghost,*a*h̬h̬$\bar{a}$*zu*, [if] (the course of the fever) is regular, [he will get well.]”

This demon *a*h̬h̬$\bar{a}$*zu* might have evolved from an original medical syndrome, as noted in Scurlock and Andersen (2005). The key point is that cases such as *a*h̬h̬$\bar{a}$*zu* might represent a creative abduction; in other words, it is a hypothesis that does not correspond to any previous syndrome or god. These syndromes, gods, demons, and so forth are not included in the handbook or in the prior knowledge of the physician. It was first necessary to create a new illness as a syndrome, which then evolved into a demon due to the established disease categories in Akkadian medicine. This creative abduction precedes the classification.

This is not a debate about whether the demon in question, which evolved from such syndromes, is authentic or not for this is something Assyriologists should establish and is not the goal of this study. I am relying on Assyriological studies in this respect. The point is that it is not unusual to find this phenomenon in medical diagnosis, as it is a fairly common expression of creative abduction in medical reasoning. Therefore, it is not surprising to encounter such cases in ancient medical diagnoses. From a methodological medical perspective, this is plausible.

As already explained, there is a clear link to the past in medical diagnosis through the uncertainty of the hypothesis. This uncertain hypothesis, or conjecture, represents the past aspect of the inference. We use medical knowledge positively when we select from it, but we also use it negatively to invent when there are no options available in our compendium. Nevertheless, it should be clarified that this does not imply that we possess all the necessary knowledge within our framework. If we did, we would be engaging with a structure of abduction akin to Inference to the Best Explanation (hereinafter ItBE). However, I am suggesting is that it is more closely related to Inference from the Best Explanation (hereinafter IfBE), in Woods’ terms,^58^. In fact, as we do not have all the possibilities at our disposal, it cannot be “to the best explanation.” The best answer we have at the moment is “from the best explanation.” In this respect, it is important to recall the somewhat ambiguous concept of explanation as discussed by Hintikka.

“[...] these facts will be [...] unknown at the time of the abduction, and even more so must the auxiliary data which help to explain them be unknown. Hence these future, so far unknown explananda, cannot be among the premises of an abductive inference” (Hintikka, (1999, p. 94).

We do not have sufficient data because if we did, there would be no abduction; instead, there would be a deduction from the data. The surprising fact remains so precisely because it is unknown, just as the hypothesis is not an affirmation but an uncertainty. These points indicate that the link to the past is established only after the fill-up movement has been completed, not before. This does not mean that the possibilities or unknown explananda are already present in the premises; rather, once the gap has been filled and linked to established knowledge, the temporal structure connects with past diagnoses. Connections can be made only after this process has been completed. Nevertheless, even though this hypothesis has been selected from a compendium, it is never certain. There is no an affirmation, but rather a conjecture. We do not have an inference to the best explanation because we lack all possible explanations. This can also be observed in the fact that there may be both selective and creative abductions. The only aspect similar to a confirmation will arise later on, once the treatment has been administered or the prediction has been verified. Even in these cases, however, there are many variables that cannot fully confirm the hypothesis.

## Conclusion

The structure of a diagnosis will depend on what time perspective it intends to adopt. In fact, it has a present-oriented focus when its purpose is to acknowledge a cluster of events associated with a disease; it is past-oriented when it attempts to isolate possible causes of a disease, and finally future-focused when it indicates a possible prognosis and an appropriate treatment for a given patient. (Chiffi, (2021, p. 10)

An understanding of the temporal structure in ancient medical science allows to gain insights into the epistemological evolution of medical reasoning through its practice. I have explained how medical diagnostic reasoning frequently features the inference structure of abduction as an explanation. This inference is a type of reasoning that cannot be reduced to deduction or induction. It preserves the schema of ignorance and is directed toward future action because it involves prognosis and treatment. The ultimate goal is to perform an action based on a hypothesis, making its primary temporal focus a future event, where the uncertainty of the hypothesis continues alongside that of the prognosis and treatment.

This has been exemplified by several Akkadian medical diagnoses and primarily corresponds to step 11 of the GW model of abduction. The most significant aspect of the Diagnostic Handbook is the direction of reasoning toward a prognosis, which is already an activation of the hypothesis under uncertainty. When the prognosis is positive, it includes treatment; conversely, when it is negative, it does not. In both cases, an action is taken in an ignorance-preserving way. In this context, the treatment is also considered a further action.

This aspect is present not only in the handbook but also in the therapeutic texts of Akkadian medicine. It is essential to view medical reasoning as a whole, taking into account the complete structure: the semiotics, disease, prognosis, and treatment. In the handbook, it can be observed how the medical trial was planned, whereas in the therapeutic texts, the trial itself can be seen.

The signs and symptoms are assessed in the present, which raises a question that cannot be answered with the knowledge at our disposal. These signs and symptoms represent the surprising fact in the inference. This point serves as the trigger for our abduction and establishes the conditions or steps of our reasoning. We do not have the answer to our question in the present; if we did, we would no longer be engaging in abduction but rather in deduction. This aspect is clearly illustrated in the Diagnostic Handbook and forms the basis of diagnosis in Akkadian medicine. Ancient physicians began with the signs and symptoms (semiotics) and conjectured a possible illness, looking for a cause.

The last point concerns the role of the past, specifically how it relates to already known maladies in our medical compendium. In Akkadian medicine, this is found in the handbook. Indeed, this aspect has two different branches. First, it is considered positively when the gap is filled with several already known hypotheses, before narrowing down the possibilities to select one. This is the most common modus operandi in the handbook. Second, it is reflected in a creative movement that expresses its relationship negatively. Examples of this include newly created demons that emerge from syndromes in Akkadian texts. When faced with a knowledge gap, something is created to fill it. If there are no viable options in our past knowledge, we generate a new one in a fill-up movement. Even if we fill it with several possibilities, including those that are created, we will still need a cut-down movement.

In this work, I have shown how medical reasoning in Akkadian medical texts can be understood as a whole when considering several texts. Some are dedicated to semiotics, diagnosis, prognosis, and sometimes treatment, whereas others focus on treatment alone. Nevertheless, to achieve a comprehensive overview of medical science in the ancient Near East, there is a need to examine medical reasoning from a broader perspective, incorporating philosophical and logical analyses that can help to visualize the structure of this reasoning.

Empirical methodology is clearly expressed in the handbook, alongside medical theory represented as hypothetical illnesses. Treatments are documented in both the handbook and the therapeutic texts. All of these elements reflect an inferential structure that is specific to ancient medicine and can be found similarly in other forms of ancient medicine. The key is to understand how ancient practitioners thought and reasoned, and for this purpose, I have used logic as a tool to study human reasoning. This methodology should help us to gain a more thorough understanding of the structure of medical practice and its evolution throughout the history of medicine.

## Footnotes

1 Many of these texts can be found in published works, such as Scurlock (2014) in English. There is also a project, BabMed, led by the Freie Universität Berlin, which publishes online copies of the medical texts along with their transliterations (see Project).

2 For an analysis and translation of the earliest diagnostic texts of Udug-Hul, see Geller (1985). It is important to note that these texts are not strictly medical. From an inferential perspective, however, they share the explanatory structure found in medical diagnoses, as will be discussed further on. The Udug-Hul series consists of magical incantations for problems that may or may not be medical in nature. For the first known medical prescription, also written in Sumerian, see Civil (1960).

3 Despite the fact that some authors call these texts a diagnostic-prognostic handbook, following Heeßel’s and Schmidtchen’s translations (Heeßel 2000, Schmidtchen 2021), I prefer diagnostic handbook. SA.GIG is the Sumerian term (lit. “sick cords”), whereas *Sakikk*$\hat{u}$ is the Akkadian title of the texts from the Diagnostic Handbook with a meaning akin to “symptoms”.

4 It should be borne in mind that the omen would have properly been considered as the prognosis, whereas the diagnosis served as the explanation (more on which below)

5 It should be stressed that in this work, no distinction is drawn between the concepts of disease, illness, and sickness as is sometimes the case in modern medicine (see Hofmann (2002)).

6 For further details on *materia medica, *see Böck (2014); Bácskay et al. (2024), plus Köcher, F. and Böck, B. The Assyrian-Babylonian Drug Lore (forthcoming).

7 This debate is not entered into here, as it has no immediate bearing on the matter at hand. For a discussion on* as*$\hat{u}$ as a physician and $\bar{a}\check{s}$*ipu* as an exorcist, see Biggs (2005). Some scholars, such as Scurlock and Andersen (2005) consider $\bar{a}\check{s}$*ipu* to be the practitioners mentioned in the Diagnostic Handbook, whereas *as*$\hat{u}$ are regarded as apothecaries. In fact, researchers still know little about how the Diagnostic Handbook was used. For more on the practical application of these texts and the fact that some as $\hat{u}$ also performed diagnoses, see Robson (2008), p. 474 and ff. It appears that the best-known exorcists from the Neo-Assyrian period made no reference to the Sag-gig series, which is also absent from the library of *Kiṣir-A*$\check{s}\check{s}$*ur* (Arbøll, 2019), p. 64. Although “the connection in *Kiṣir-A*$\check{s}\check{s}$*ur*’s tablets to Sag-gig is evident,” the correlation between Sag-gig and therapeutic material seems inconsistent to some authors, as noted in Arbøll (2019), p. 64. Arbøll also suggests that “*Kiṣir-A*$\check{s}\check{s}$*ur* might well have been taught according to principles differing from those appearing in the Sa-gig series”, p. 65.

8 These concepts, introduced by Magnani (2001), are contextual and also take into account the past from an eco-cognitive perspective. For details, see Magnani (2015a), p. 290 and ff. It warrants noting that a criterion of plausibility, minimality, or consistency is not necessary for the hypothesis to be the best or even adequate.

9 For a detailed description of abduction as an explanation and deductions as predictions or prognoses in medicine, see Barés Gómez and Fontaine (2022) and the following sections.

10 There are also several examples that refer to illnesses, such as “Sick Lungs,” but to the best of my knowledge, they are not often found in the Diagnostic Handbook (more on this aspect below).

11 This is a reformulation of the Avalos structure proposed by Heeßel 2000, p. 41. The English translation is my own. See also Schmidtchen (2021), p. 54 and ff.

12 For a complete analysis of Akkadian medical diagnosis as an abductive inference, see Barés Gómez (2018).

13 It should be taken into account that there have been many problems in defining abduction, while some authors have considered it to be a form of induction. Here, abduction is regarded as a type of inference differing from induction and deduction, as noted in Barés Gómez and Fontaine (2022); Gabbay and Woods (2005); Hintikka (1999); Magnani (2001, 2015a, 2015b, 2017, 2019a); Park (2015); Woods (2013). For further information on the problems associated with abduction and its interpretation in the philosophy of science, see Plutynski (2011).

14 See Heeßel 2000; Scurlock 2014.

15 The text is from the Diagnostic Handbook, tablet 33, line 93. The English translation is from Scurlock (2014). p. 239. See also AOAT 43.357, the abbreviation AOAT deriving from Heeßel 2000, p. 357 and ff. The AOAT translation is “[Wenn sein Gesicht gelb - gr]ünlich wird, das Innere seiner Augen gelb-grünlich wird und seine Zungenwurzel schwarz wird:[a] h̬h̬[$\bar{a}$*-zu *ist ihr Name].” Similarly in Labat (1951), TDP 9/13: “Si son visage est jaune, l’intérior de ses yeux, jaune, et la racine de sa langue, noire: (c’est), le démon Ah̬h̬ $\bar{a}$*zu.” *See also chapter 3 in Scurlock and Andersen (2005); Schmidtchen (2021), p. 415. and BAM 578, IV, 43-45 and 26. Usually, this demon is associated with jaundice, namely, a symptom of jaundice or a demon personifying the disease itself, as discussed in Adamson (1993) and the final chapter of this work.

16 See also Magnani (2001).

17 Sometimes there is also DIŠ GIG... ( if the ill person...).

18 Although this is the usual form, there are also others, as seen in example 1. For a complete analysis of the verbs and forms, among other things, see Heeßel 2000 and Schmidtchen (2021).

19 Usually, this cause—whether it be a punishment, illness, or divine sender—appears as the “Hand of the God.” For an analysis, see Heeßel (2007).

20 The fact that he is not in a state of wellbeing derives from the contrast between the concept of illness and wellbeing, for which reason medical texts usually describe both. In Akkadian there are physiognomic omens, *Alamdimm*$\hat{u}$. For these texts, see Böck (2000).

21 As Barés and Fontaine have argued in Barés Gómez and Fontaine (2022), there is really no such thing as a partial abduction in medical reasoning. In any case, as action is taken—even clinically testing it is an action—so the hypothesis is always used in step 11. In this connection, I would like to thank one of the anonymous referees for suggesting the role of extispicy in Akkadian medical thinking Heeßel 2018. The study of the liver and other animal organs as a form of divination seems to have served as a kind of verification sometimes functioning as inductive proof and sometimes as a prognosis as a form of post-abduction verification. Of course, this is not a verification in modern scientific methodology, but does occupy a place in the inferential structure. This point should be analyzed more comprehensively in future studies.

22 For a complete analysis, see Woods (2013), p. 364 and ff.

23 For a detailed analysis, see Arbøll (2019), p. 169 and ff.

24 The text is from the Diagnostic Handbook, tablet 22, line 16. The English translation is from Scurlock (2014), p. 189. Another translation can be found in AOAT 43, 253, while the abbreviation AOAT is from Heeßel 2000, p. 253 and ff. The original German is as follows: “Wenn bei seinem Sprechen Speichel läuft, sein Gesicht voll mit Gelbsucht ist, seine Eingeweide sich entleeren: Hand eines Eides, er wird sterben.”

25 For an analysis of uncertainty and treatment in modern medical prognosis, see Chiffi and Andreolotti (2022) and Campaner and Sterpetti (2022), respectively.

26 The text is from the Diagnostic Handbook, tablet 40, line 29. The English translation is from Scurlock (2014), p. 264. Another translation can be found in TDP Labat (1951). The original French translation is as follows: “Si, le bébé, ses chairs se marquent de jaune, si ses entrailles son entravées, si ses mains et ses pieds sont enflammés, s’il a beaucoup de fièvre: il est malade des poumons); main du dieu; il guérira.”

27 See Schmidtchen (2021), p. 157 and ff.

28 See Heeßel 2000, p. 63 and ff.

29 For a detailed study of fevers and temperatures, see Stol, M. “Fevers in Babylonia” in Finkel & Geller (2007) and Bácskay (2018).

30 The text is from the Diagnostic Handbook, tablet 3, line 79-80. The English translation is from Scurlock (2014), p. 22. Another translation can be found in Schmidtchen (2021) which should be contrasted with 3.52 and 3.68. Schmitdtchen translated this as “he will grow old (lit.: his days will be lengthened) and die,” instead of Scurlock’s rendering, “he will get well”. Other translations can be found in Labat (1951), p. 29, line 82. Labat talks about *Lama*$\check{s}$*tu* rather than *A*h̬h̬$\bar{a}$*zu.*

31 See the reference and common aspects of these two medicines already addressed by Labat (1951), Geller and Stol (Horstmanshoff et al., 2004) and Heeßel 2000.

32 As they have the structure of a conditional, they resemble omens, but are actually explanations followed by predictions.

33 Even though the Diagnostic Handbook contains some treatments (e.g., tablet 31 which will be discussed further on), this is infrequent.

34 For the structure of the diagnostic and therapeutic texts, see Geller (2010), p. 40. See also (Stol 1991-92).

35 See also Steinert et al. (2018) and the chapter entitled, The Assur Medical Catalogue (AMC), Ulrike Steinert (ed).

36 The text is BAM 578. The abbreviation of BAM is from (BAM) and the English translation from Scurlock (2014), p. 526. See also note 32 in Adamson (1993) and (Stol 1991-92), p. 53. Another translation can be found in Johnson and Simkó (2024), pp. 200-201: “If someone’s body is yellow (*aruq*), his face is yellow and black (*ṣalim*) (and) the root of his tongue is black, its name is *a*h̬h̬$\bar{a}$*zu -* jaundice. You pound *pizallur(t)u rabītu* (“big geko”) from the steppe, he drinks (*i*$\check{s}$*atti*) it in beer, (and then) the *a*h̬h̬$\bar{a}$*zu - *jaundice in his belly will come straight out (*i*$\check{s}\check{s}$*eram*).”

37 There are some cases with negative prognoses that the physician tries to treat in the therapeutic texts, but only to try to change them. For a complete explanation, see (Stol 1991-92).

38 It should be taken into account that the knowledge we have about the use of the handbook is still poor.

39 See also an analysis of pre-Babylonian texts from Nippur in the eighteenth century BC which refer to unknown causes, in contrast with the first-millennium BC texts from the Diagnostic Handbook which specify the causes in Geller (2006).

40 This assessment can be found in Johnson and Simkó (2024), p. 190, n. 531, and p. 200, n. 558. See also Labat (1951), pp. 72-73 and Heeßel 2000, p. 357.

41 See also an analysis of the Hippiatric texts of Ugarit in Barés (2021)

42 It warrants noting that reference is not being made to experiments as in modern science with a proper methodology, but an approach to the experience.

43 See Stol (1991-92) (xxx); Geller (2010), p. 90 and ff. and Johnson and Simkó (2024).

44 The prescriptions are not used randomly, but rather as guidelines and have been tested several times previously. For further details, see Steinert (2015) and Arbøll (2019), p 192 and ff.

45 For this approach, see also Barés Gómez and Fontaine (2022).

46 For further details, see Böck (2014), chapter 3.

47 The English translation is from Böck (2014). There is knowledge about plants in the “List of simple drugs” and URU.AN.NA: *ma*$\check{s}$*takal.* For a complete study of these plants for jaundice* bu’*$\check{s}\bar{a}$*nu* and *li*$\check{s}\bar{a}$*n kalbi*, see Böck (2014). See also Bácskay et al. (2024) for Ancient Mesopotamian pharmacopoeia.

48 This may also be why some researchers claim that the *as*$\hat{u}$ was more akin to a pharmacologist than a doctor. In this respect, see Scurlock (2014)

49 The text is from the Diagnostic Handbook, tablet 31, line 1-2. The English translation comes from Scurlock (2014), p. 227. Another translation in German SA.GIG 31/1-2, AOAT is from Heeßel 2000, p. 342 and ff.

50 For a detailed analysis of this observation as a scientific characteristic of medical reasoning in Akkadian medicine, see Heeßel (2024a). The medical omens not only involve the prognostic-omens, but also the abduction-explanation step. Besides, they are grounded in empirical facts with a systematic observation corresponding to the surprising fact/facts.

51 This aspect of the complete inferential structure could explain suggestions, such as that made by Böck (2014), for whom the definition of a disease also influences treatment. Once the hypothetical disease has been determined as the affliction of a god X, the plants associated with that divinity are used.

52 For an analysis of the risks and evaluation of a eco-cognitive model of this in medicine, see Barés (2021).

53 The text is from the Diagnostic Handbook, tablet 13, lines 75-76. The English translation is from Scurlock (2014), p. 113. Another translation can be found in Schmidtchen (2021) p. 521 and ff. See also Labat (1951), pp. 118-119, line 17.

54 Other differential diagnoses are also mentioned in SA.GIG, *Sakikk*$\hat{u}$ 10/32-33. However, it is not clear whether they are truly differential or merely a compilation of different traditions. See Heeßel 2000, p. 57 and Schmidtchen (2021), p. 159.

55 For a detailed explanation, see Scurlock and Andersen (2005), pp. 505-506. It is important to note that some of the origins are fairly hotly debated in Assyriological studies. For example, in the case of *Lama*$\check{s}$*tu*, also referred to as the “baby-snatching demon,” authors such as Scurlock and Kinnier Wilson suggest it may be a demon created from the signs and symptoms of an illness, which could be typhoid or typhus. Other authors believe it might be associated with a different kind of illness or fever, such as liver disease, whereas others contend that it cannot be linked to a single illness. I will not enter into this debate; for further analysis, see the chapter entitled,* Lama*$\check{s}$*tu* - Agent of a Specific Disease or Generic Destroyer of Health?. W. Farber in Finkel & Geller (2007).

56 It is important to stress that I am not proposing any theory regarding the creation of this demon. From an inferential point of view, an illness can be created at some point; thus, it is possible that the name or syndrome was established before the compilation, leading to the demon originating from the syndrome. This phenomenon occurred when a new illness emerged that had not yet been included in the compendium. This inferential step could explain why the syndrome became a demon, rather than vice versa, as is typically described in Akkadian medicine. Nevertheless, the origins of this syndrome are something that Assyriologist must establish using the available data.

57 The text is from the Diagnostic Handbook tablet 3, lines 79-80. The English translation is from Scurlock (2014), p. 22. See also Schmidtchen (2021), line 68, pp. 253-260.

58 It should be taken into account that I do not assume abduction to IBE (in this work ItBE) or to IfBE (Woods, 2013) but consider IfBE might characterize some abductions. I do not consider IfBE to define abduction. For abductions which are not IfBE, see Barés Gómez and Fontaine (2020); Gabbay and Woods (2005); Magnani (2001); Woods (2013); Magnani (2017).
